# Protective Effects of COG133 on Carbon Tetrachloride‐Induced Acute Liver Injury: Modulation of Inflammation, Apoptosis and Sphingolipid Metabolism

**DOI:** 10.1111/jcmm.70677

**Published:** 2025-06-21

**Authors:** Mutay Aslan, Bürke Çırçırlı, Aleyna Öztüzün, Hazal Tuzcu, Çağatay Yılmaz, Tuğçe Çeker, Gülsüm Özlem Elpek

**Affiliations:** ^1^ Department of Medical Biochemistry Akdeniz University Faculty of Medicine Antalya Turkey; ^2^ Department of Medical Biotechnology, Institute of Health Sciences Akdeniz University Antalya Turkey; ^3^ Department of Pathology Akdeniz University Faculty of Medicine Antalya Turkey

**Keywords:** acute hepatotoxicity, apoptosis, carbon tetrachloride, COG133, inflammation, sphingolipids

## Abstract

Acute liver hepatotoxicity, characterised by inflammation, apoptosis and metabolic dysfunction, is often caused by drug‐induced toxic events. This study evaluated the protective effects of COG133, a synthetic peptide derived from apolipoprotein E (ApoE), against carbon tetrachloride (CCl_4_)‐induced liver damage, focusing on inflammation, apoptosis and sphingolipid metabolism. An acute hepatotoxicity model was established in rats utilising CCl_4_, with co‐administration of COG133 at varying doses. Histological analyses, immunostaining, messenger RNA (mRNA)/protein quantification, flow cytometry and mass spectrometry were employed to assess necroinflammation, apoptosis and sphingolipid levels. Cell viability assays and morphological evaluations were conducted on rat hepatocytes and hepatic stellate cells (HSC‐T6) to evaluate the protective effects of COG133. COG133 reduced liver damage, necroinflammation and apoptosis, restoring cell viability and lowering markers of inflammation, fibrosis and oxidative stress, including tumour necrosis factor‐alpha (TNF‐α), nuclear factor kappa‐B (NF‐κB), inducible nitric oxide synthase (NOS2), interleukin‐1 beta (IL‐1β), transforming growth factor‐beta (TGF‐β) and collagen type I (Col‐1). Immunostaining and molecular analyses confirmed these effects. Sphingomyelin (SM) and sphingosine‐1‐phosphate (S1P) levels were partially restored, while ceramide (CER) levels remained reduced in COG133‐treated groups. COG133 protects against CCl_4_‐induced liver injury by reducing inflammation, apoptosis and morphological damage, with partial restoration of sphingolipid metabolism. These findings support its potential as a novel therapeutic agent for acute liver injury.

**Abbreviations**
ApoE: apolipoprotein E
CCl_4_
: carbon tetrachloride
CER: ceramide
COG: cogniscience ApoE mimetic peptide
COG133: ApoE‐mimetic peptide
Col‐1: collagen type I
CTCF: corrected total cell fluorescence
DAPI: 4′,6‐diamidino‐2‐phenylindole
DMEM: Dulbecco's Modified Eagle's Medium
DMSO: dimethyl sulfoxide
ELISA: enzyme‐linked immunosorbent assay
HSCs: hepatic stellate cells
IL‐1: interleukin‐1
IL‐1β: interleukin‐1 beta
LDL: low‐density lipoprotein
LDLR: low‐density lipoprotein receptor
mRNA: messenger RNA
NF‐κB: nuclear factor kappa‐B
NGS: normal goat serum
NOS2: inducible nitric oxide synthase
PI: propidium iodide
RT‐PCR: reverse transcription polymerase chain reaction
S1P: sphingosine‐1‐phosphate
SMs: sphingomyelins
TGF‐β: transforming growth factor‐beta
TNFR1: tumour necrosis factor‐alpha receptor‐1
TNF‐α: tumour necrosis factor‐α
TUNEL: terminal deoxynucleotidyl transferase dUTP nick end labelling

## 1 Introduction

Drug‐induced toxic events are defined as one of the most common causes of liver damage. This is because the liver is the primary organ for the metabolism of many drugs or chemical agents [1]. The causes of toxic hepatitis include drugs, natural toxic agents and chemical substances [2]. In this study, CCl_4_ was used to create an acute hepatotoxicity model in rats [3]. Today, CCl_4_ has proven to be a highly useful experimental model for investigating acute hepatotoxic effects. It consistently induces liver damage in many species, including non‐human primates and humans [4]. The type and extent of liver damage encompass a wide range of effects, and the severity of lesions can be increased or decreased by various beneficial or harmful interventions [4].

Most of the inflammatory and molecular events following acute hepatotoxicity are mediated not only by hepatocytes but also by two types of non‐parenchymal liver cells: Kupffer cells and stellate cells [5]. Once activated, Kupffer cells, the resident macrophages of the liver, secrete TNF‐α, nitric oxide, IL‐1, IL‐6 and IL‐10 [6]. Stellate cells, upon activation by cytokines, exhibit a typical acute‐phase response, adopt a fibroblast‐like appearance, secrete nitric oxide and begin to overproduce type I collagen, thereby promoting hepatic fibrosis [6].

TNF‐α released because of acute hepatotoxicity leads to the activation of the NF‐κB pathway via tumour necrosis factor‐alpha receptor‐1 (TNFR1) [7]. The primary mechanism of the NF‐κB pathway activation involves the activation of a multi‐subunit IκB kinase (IKK) complex. The IKK complex consists of two catalytic subunits, IKKα and IKKβ, and a regulatory subunit called IKKγ. IKK, activated via the TNFR1 receptor, phosphorylates IκBα on two N‐terminal serine residues [8]. As a result of phosphorylation, the translocation of NF‐κB subunit p50/p65 dimers to the nucleus occurs [7]. In the nucleus, active NF‐κB induces the expression of NOS2, IL‐1, IL‐6 and IL‐10 [9]. Additionally, TNF‐α released during acute hepatotoxicity can stimulate the activity of neutral sphingomyelinase via TNFR1, causing changes in CER levels derived from SM [10]. Changes in the sphingolipid composition of tissues and cells may alter membrane structure and organisation.

Apolipoprotein E is an exchangeable apolipoprotein associated with high‐density lipoprotein, very low‐density lipoprotein and low‐density lipoprotein (LDL) remnants [11]. It is primarily synthesised by hepatocytes and macrophages and plays a critical role in regulating plasma cholesterol levels [12]. A hinge region divides the two structural domains of ApoE. The receptor binding region (amino acids 134–150 and Arg‐172) is in the N‐terminal domain (amino acids 1–191), which also forms a four‐helix antiparallel bundle. The primary lipid binding site (amino acids ∼244–272) is in the C‐terminal domain (amino acids ∼225–299) [13]. Both the lipid‐associated and receptor‐binding domains of ApoE have been shown to be essential for lipoprotein binding and clearance [14].

Numerous synthetic peptides have been designed based on the structure of ApoE [15]. Researchers have incorporated alpha‐aminoisobutyric acid groups into residues 133–149 of ApoE to create chimeric peptides with increased helicity. These peptides, known as Cogniscience ApoE mimetic peptides (COGs), have been reported to exhibit significant anti‐inflammatory effects [16]. In our study, the COG 133 peptide (ApoE 133–149 amino acid region) was used. The ApoE mimetic peptide COG 133 binds to the low‐density lipoprotein receptor (LDLR), inhibits TNF‐α secretion, reduces NF‐κB phosphorylation and suppresses the inflammatory response [17]. LDLR is present in rat hepatocytes, Kupffer cells and stellate cells [18, 19, 20].

In this study, we created a model of acute hepatotoxicity in rats and liver cell cultures and examined the protective mechanism of COG 133 in vivo and in vitro, respectively. To the best of our knowledge, there are no studies examining the effects of ApoE mimetic peptides on acute hepatotoxicity. The hypothesis of this study was that COG 133 could suppress the inflammation in the liver caused by acute hepatotoxicity. The main objective was to establish acute toxicity in rat liver in vivo and rat liver cell cultures in vitro and to apply COG 133 in the experimental models created. The effect of COG 133 on the TNF‐α signalling pathway, liver destruction, and sphingolipid metabolites was also determined. The selection of two COG133 doses in this study was strategically based on previous in vivo studies in mice [17], ensuring translational relevance while maintaining experimental rigour. Moreover, primary hepatocytes and hepatic stellate cell lines (HSC‐T6) were employed as established in vitro systems, allowing for the mechanistic exploration of hepatoprotective effects in a controlled environment. These complementary models are widely accepted for evaluating liver injury and therapeutic interventions before advancing to more complex systems [21].

## 2 Materials and Methods

### 2.1 Experimental Animal Model

Four experimental groups were established: Group 1 served as the control; Group 2 received CCl_4_; Group 3 received 1 μM ApoE COG133 peptide with CCl_4_; and Group 4 received 3 μM COG133 with CCl_4_. Each group consisted of eight male Wistar rats (4 weeks old, weighing 180–200 g). The study was approved by the Animal Experiments Local Ethics Committee (Decision No: 30‐Date: 09.03.2023). Animals were fasted overnight for 12 h prior to the induction of anaesthesia and blood collection.

CCl_4_ (99.5%, molecular weight: 153.82 g/mol; Sigma Aldrich, St. Louis, MO, USA) was given as a single subcutaneous injection of 1 mL/kg body weight in a 1:1 mixture with olive oil daily for 4 days to induce acute hepatotoxicity [22]. Rats were euthanized 24 h after the final dose. Euthanasia was conducted via intraperitoneal injection of ketamine (80 mg/kg) and xylazine (12 mg/kg). Blood samples were collected from the inferior vena cava, and the liver was perfused with 0.9% NaCl and harvested.

COG133 peptide was administered intraperitoneally at doses of 1 and 3 μM, determined based on a prior study showing anti‐inflammatory effects in mice [17]. A stock solution of 461 μM was prepared by dissolving 1 mg of COG133 (MW = 2169.73 g/mol; AdooQ Bioscience, Irvine, USA) in 1 mL distilled water. Final concentrations were achieved by diluting the stock. COG133 was administered twice daily (10 μL/g body weight) for 4 days, with CCl_4_ given 1 h after the first dose. Based on calculations, a 200‐g rat received a daily dose of 8.68 μg COG133 peptide at a concentration of 1 μM, while the daily dose at a concentration of 3 μM was calculated as 26.04 μg. Control rats received 400 μL of saline‐olive oil mixture and 2 mL of sterile water intraperitoneally for 4 days. All rats were euthanized on day 5, and samples were collected as described.

### 2.2 Liver Tissue Histopathological Evaluation

Liver tissues were fixed in 10% neutral buffered formalin and sectioned into 5 μm slices utilising a microtome. Haematoxylin and eosin staining was conducted, and slides were analysed under a light microscope (Olympus IX81, Tokyo, Japan) by a blinded pathologist. The Ishak modified hepatic activity index was used to score necroinflammation, assessing confluent/lytic necrosis (0–6), portal inflammation (0–4) and interface hepatitis (0–4) [23].

### 2.3 Tissue Immunohistochemical Staining

The Dako Omnis Closed System Immunohistochemistry Staining Device (Agilent Technologies, Santa Clara, USA) was used for immunohistochemical staining. Using rabbit polyclonal antibodies, the primary antibody application was carried out for 60 min at 25°C. The primary antibodies that were utilised were anti‐TNF‐α (1:200, #E‐AB‐33121, Elabscience; Houston, Texas, USA), anti‐TGF‐β (1:300, #AP14911, BT Lab, Bioassay Technology Laboratory; Shanghai, China), anti‐Col‐1 (1:200, #MBS2114290, MyBioSource, San Diego, CA, USA), anti‐NF‐KB p65 (1:200, #ab16502, Abcam, Cambridge, MA, USA), anti‐IL‐1β (1:200, #AP04470, BT Lab, Bioassay Technology Laboratory; Shanghai, China), and anti‐NOS2 (1:100, #610332, BD Biosciences Pharmingen, San Jose, California, USA). For negative control groups, the staining protocol was applied by adding 5% normal goat serum (NGS) instead of the primary antibody. The streptavidin‐peroxidase conjugate was utilised to conduct the reaction, and a secondary antibody labelled with biotin was employed. The percentage of positively stained cells was used to calculate the immunostaining score [23]. The percentage score for positively stained cells was 0 = < 5% cells/40× magnification area; 1 = 5%–30% cells/40× magnification area; 2 = 30%–50% cells/40× magnification area; and 3 = > 50% cells/40× magnification area.

### 2.4 Rat Hepatocyte and Stellate Cell Cultures

The rat hepatocyte cell line (catalogue #r033‐BRL, iCell; Shanghai, China) was purchased commercially. The HSC‐T6 rat hepatic stellate cell line was commercially obtained (catalogue #SCC069, Sigma‐Aldrich‐Merck Millipore, Molsheim, France). Rat hepatocyte (BRL) and HSC‐T6 were cultured in Dulbecco's Modified Eagle's Medium (DMEM) and a 1:1 mix of Ham's F‐12/DMEM, respectively. Media were supplemented with fetal bovine serum (10%), penicillin (100 U/mL), streptomycin (100 μg/mL), sodium bicarbonate (3.7 g/L), sodium pyruvate (1%) and amphotericin‐B (1%). Cells were grown in a humidified incubator at 37°C with 5% CO_2_ and passaged at 80%–90% confluence utilising trypsin ethylenediaminetetraacetic acid. Cell passages 0–10 were used throughout the study.

### 2.5 Dose‐Time Dependent Effect of CCl_4_ and COG133 on Cell Viability

CCl_4_ was prepared in culture medium containing 0.25% dimethyl sulfoxide (DMSO) at a final concentration of 0.4% (v/v) [24]. COG133 stock (461 μM) was diluted to final concentrations of 0.02–20 μM [15]. Cells were treated with CCl_4_ for 30 min to 24 h or with COG133 for 24 h. Cell viability was evaluated utilising the MTT assay. Absorbance was measured at 570 nm (background at 690 nm subtracted) utilising a microplate reader (Bio‐Tek Instruments Inc., Vermont, USA). Results were expressed as a percentage of the control group.

### 2.6 Staining of Cells by Immunofluorescence

Approximately, 100,000 cells per well were seeded onto 8‐well chamber slides (Merck Millipore, Cork, Ireland) and incubated overnight. Cells were treated as per experimental protocols and fixed with 4% paraformaldehyde for 10 min. Permeabilization was done utilising 0.2% Triton X‐100, followed by blocking with 5% NGS. Primary antibodies (anti‐TNF‐α, anti‐TGF‐β, anti‐Col‐1, anti‐NF‐κB p65, anti‐IL‐1β and anti‐NOS2) were incubated overnight at 4°C, and secondary Alexa Fluor‐488‐conjugated antibodies were applied for 45 min at room temperature. Nuclei were stained with DAPI. Images were acquired utilising a fluorescence microscope (Olympus BX61, Tokyo, Japan). Fluorescence intensity was quantified utilising NIH ImageJ 1.53e software, and the corrected total cell fluorescence (CTCF) was calculated as CTCF = Integrated density − (Area of cell × Average background readings).

### 2.7 Reverse Transcription Polymerase Chain Reaction (RT‐PCR)

All mRNA sequences were sourced from the Rat Genome Database (https://rgd.mcw.edu/rgdweb/homepage/). Primers and probes were designed utilising the OligoYap 9.0 software (SNP Biotechnology R&D Ltd., Ankara, Turkey). The primers and probes used are shown in Table S1. Real‐time PCR analysis was conducted utilising the One‐run RT PCR kit (SNP Biotechnology R&D Ltd., Ankara, Turkey). Total RNA was extracted utilising a commercial kit and dissolved in TE buffer. RNA purity was evaluated by spectrophotometry, with an acceptable 260/280 ratio of ~2. cDNA synthesis and real‐time PCR analysis were conducted utilising the One‐Run RT‐PCR kit (SNP Biotechnology R&D Ltd., Ankara, Turkey). Optimal primer and probe concentrations were determined to minimise Ct values. Amplification conditions were 42°C for 10 min, 90°C for 5 s and 60°C for 45 s, for 45 cycles. Relative mRNA levels were calculated utilising the 2^(−ΔΔCt)^ method, normalised to 18S rRNA.

### 2.8 Enzyme‐Linked Immunosorbent Assay (ELISA)

Protein levels of TNF‐α, NF‐κB, NOS2, IL‐1β and TGF‐β were measured utilising ELISA kits (Elabscience, Houston, TX, USA; Bioassay Technology Laboratory, Zhejiang, China). Standards and samples were added to 96‐well plates and incubated with detection antibodies. After washing, a substrate solution was added, and absorbance was measured at 450 nm utilising a microplate reader. Protein concentrations were calculated based on standard curves. Curve fitting and interpolation were conducted utilising GraphPad Prism software to analyse sample concentrations on nonlinear standard curves. Results were reported as per mg of tissue or cell protein.

### 2.9 Apoptosis

TUNEL staining was conducted utilising the TUNEL In Situ Apoptosis Kit (Elabscience, Houston, TX, USA) to detect apoptotic cells. Rat hepatocytes and stellate cells were seeded at 100,000 cells per well on chamber slides, fixed with paraformaldehyde and incubated with TdT enzyme at 37°C for 25 min. After applying a labeling solution, slides were stained with DAPI and visualised utilising a fluorescence microscope.

Apoptosis was further analysed utilising a FITC‐Annexin‐V/PI kit. Treated cells were stained with Annexin‐V and PI and analysed utilising flow cytometry (FACS Canto II; BD Biosciences). Data were processed with BD FACS Diva software, reporting apoptotic cell percentages.

### 2.10 Ceramide and Sphingomyelin Analysis

Ceramide and SM levels were analysed utilising a Shimadzu LC/MS–MS system (LCMS‐8040) connected to an ultra‐fast liquid chromatography system (LC‐20 AD UFLC XR, Shimadzu Corporation, Japan). The procedure was previously described in detail [25, 26]. Samples were prepared with chloroform: methanol extraction and analysed utilising an XTerra C18 HPLC column under gradient elution. Specific MRM transitions were used for quantification, with linear calibration curves between 39 and 625 ng/mL.

### 2.11 Protein Analysis

Protein concentrations in tissue and cell lysates were determined at 595 nm utilising the Bradford method, with BSA as the standard (Pierce Chemical Company, Rockford, IL).

### 2.12 Statistical Analysis

Statistical analyses were conducted utilising GraphPad Prism 8.4.3 (GraphPad Software, San Diego, California, USA) or SigmaPlot 15 for Windows (Systat Software Inc., Palo Alto, CA, USA). Detailed results of the statistical analyses are provided in the figure legends. First, a normality test was conducted utilising the statistical program. A test that passed demonstrated that the data came from a normally distributed population and would exhibit the expected pattern. If the sample data were not normally distributed, the software conducted a non‐parametric test, meaning the normality test failed. One‐Way ANOVA (Analysis of Variance) or Kruskal‐Wallis One‐Way ANOVA were used to compare the experimental groups. Post hoc tests, which are multiple comparison techniques, were utilised to identify the specific groups that differed from one another when a statistically significant difference was found. A *p*‐value of < 0.05 was considered statistically significant.

## 3 Results

### 3.1 Acute Liver Hepatotoxicity

To assess the impact of COG133 on acute liver injury, changes in body weight and liver morphology were first evaluated. Body weights significantly increased by day 5 following hepatotoxicity model induction (Figure S1A), although liver‐to‐body weight ratios showed no significant differences among the groups (Figure S1B). Macroscopic liver images (Figure S1C) demonstrated marked damage in rats treated with carbon tetrachloride (CCl_4_), characterised by pallor and textural changes. In contrast, co‐treatment with COG133 markedly reduced these visible lesions.

Histological evaluation further confirmed these protective effects. Haematoxylin and eosin (H&E) staining (Figure S1D) revealed extensive necrosis and steatosis in CCl_4_‐treated rats, whereas COG133 administration (at both 1 and 3 μM) significantly reduced necroinflammatory changes. Quantitative scoring (Table S2) supported these observations, showing lower necroinflammation scores in the COG133 groups compared to the CCl_4_ group (*p* < 0.05).

### 3.2 Protective Effects of COG133 on Cell Viability

**FIGURE 1:**
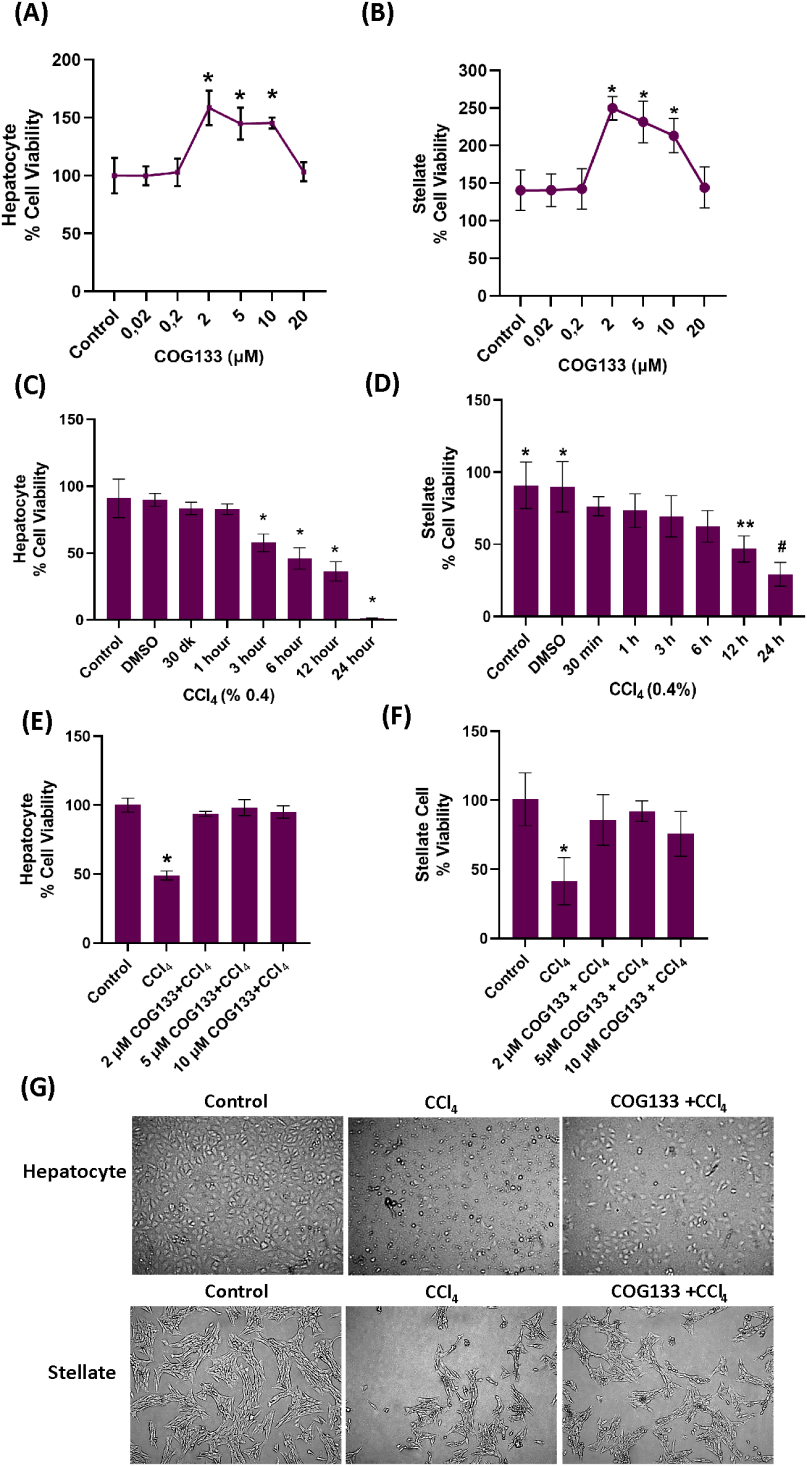
(A) 24‐h cell viability analysis of COG133 (μM) administration in rat hepatocyte cells. Data represents 10–12 separate measurements, and values are mean ± SD. Statistical analysis was conducted by one‐way ANOVA analysis. The difference between groups was determined by Tukey's test. *p < 0.001 compared to control, 0.02, 0.2, and 20 μM groups. (B) 24‐h cell viability analysis of COG133 (μM) administration in rat hepatic stellate cells. Data represents 6–8 separate measurements, and values are mean ± SD. Statistical analysis was conducted by one‐way ANOVA analysis. The difference between groups was determined by Tukey's test. *p < 0.001, compared to the control, 0.02, 0.2, and 20 μM groups. (C) Cell viability analysis of CCl_4_ (0.4%) administration in rat hepatocyte cells at different time points. Cells treated with DMSO (0.25%). Data represent 9–12 separate measurements and values are mean ± SD. Statistical analysis was conducted by one‐way ANOVA analysis. The difference between groups was determined by Tukey's test. *p < 0.05, compared to control, 30 min and 1‐h groups. (D) Cell viability analysis of CCl_4_ (0.4%) administration in rat HSCs in different time points. Data represents 6–12 measurements, and values are mean ± SD. Statistical analysis was conducted by one‐way ANOVA analysis. The difference between groups was determined by Tukey's test. *p < 0.05, vs. 3‐, 6‐, 12‐, and 24‐h groups. **p < 0.05, vs. control, DMSO, 30 min, 1‐, 3‐, and 24‐h groups. ^#^ *p* < 0.05, compared with all groups. (E) Cell viability analysis of 24‐h COG133 (2–10 μM) and 6‐h CCI_4_ (0.4%) administration in rat hepatocyte cells. CCI_4_ was given 18 h after COG133 administration. The data represents 10–12 separate measurements, and values are mean ± SD. Statistical analysis was conducted with Kruskal‐Wallis test. The difference between groups was determined by Dunn's analysis. *p < 0.05, compared to all groups. (F) Cell viability analysis of COG133 (2–10 μM) in rat HSCs for a total of 24 h. CCl_4_ (0.4%) was given 12 h after COG133 administration. The data represents 6–8 separate measurements, and values are mean ± SD. Statistical analysis was conducted by one‐way ANOVA analysis. The difference between groups was determined by Tukey's test. *p < 0.001, compared to all groups. (G) Light microscope image of hepatocytes and stellate cells (10× magnification). Hepatocyte CCl_4_ group, cells treated with CCl_4_ (0.4%) for 6 h; Hepatocyte COG133 + CCl_4_ group, cells to which CCl_4_ (0.4%) was administered 18 h after 2 μM COG133 administration. The total incubation period was 24 h. Stellate cell CCl_4_ group, cells treated with CCl_4_ (0.4%) for 12 h; Stellate cell COG133 + CCl_4_ group, CCl_4_ (0.4%) given 12 h after 5 μM COG133 administration. The total incubation period was 24 h. In both cell groups, COG133 administration was observed to reduce morphological deterioration due to CCl_4_ (0.4%) administration.

The effect of COG133 on hepatocyte and stellate cell viability was next evaluated using MTT assays. In hepatocytes, treatment with 2 μM COG133 significantly enhanced cell viability compared to untreated controls and other concentrations (Figure 1A). Exposure to 0.4% CCl_4_ for 3 h or more induced a significant reduction in cell viability (Figure 1C). Pre‐incubation with 2 μM COG133 for 24 h, followed by CCl_4_ challenge, mitigated this cytotoxicity, with a significant improvement in viability compared to the CCl_4_‐only group (Figure 1E).

In hepatic stellate cells (HSC‐T6), a similar pattern was observed. COG133 at concentrations of 2–10 μM significantly enhanced viability after 24 h compared to both lower (0.02 and 0.2 μM) and higher (20 μM) doses (Figure 1B). CCl_4_ exposure for 12 h significantly reduced viability (Figure 1D), but pre‐treatment with 2, 5, or 10 μM COG133 restored viability (Figure 1F). Morphological assessments (Figure 1G) showed that COG133 attenuated CCl_4_‐induced morphological disruptions such as cell rounding, separation and shrinkage in both cell types. Collectively, these results demonstrate a clear dose‐ and time‐dependent cytoprotective effect of COG133 against CCl_4_‐induced injury.

### 3.3 Modulation of Inflammatory, Fibrotic and Oxidative Markers

**FIGURE 2:**
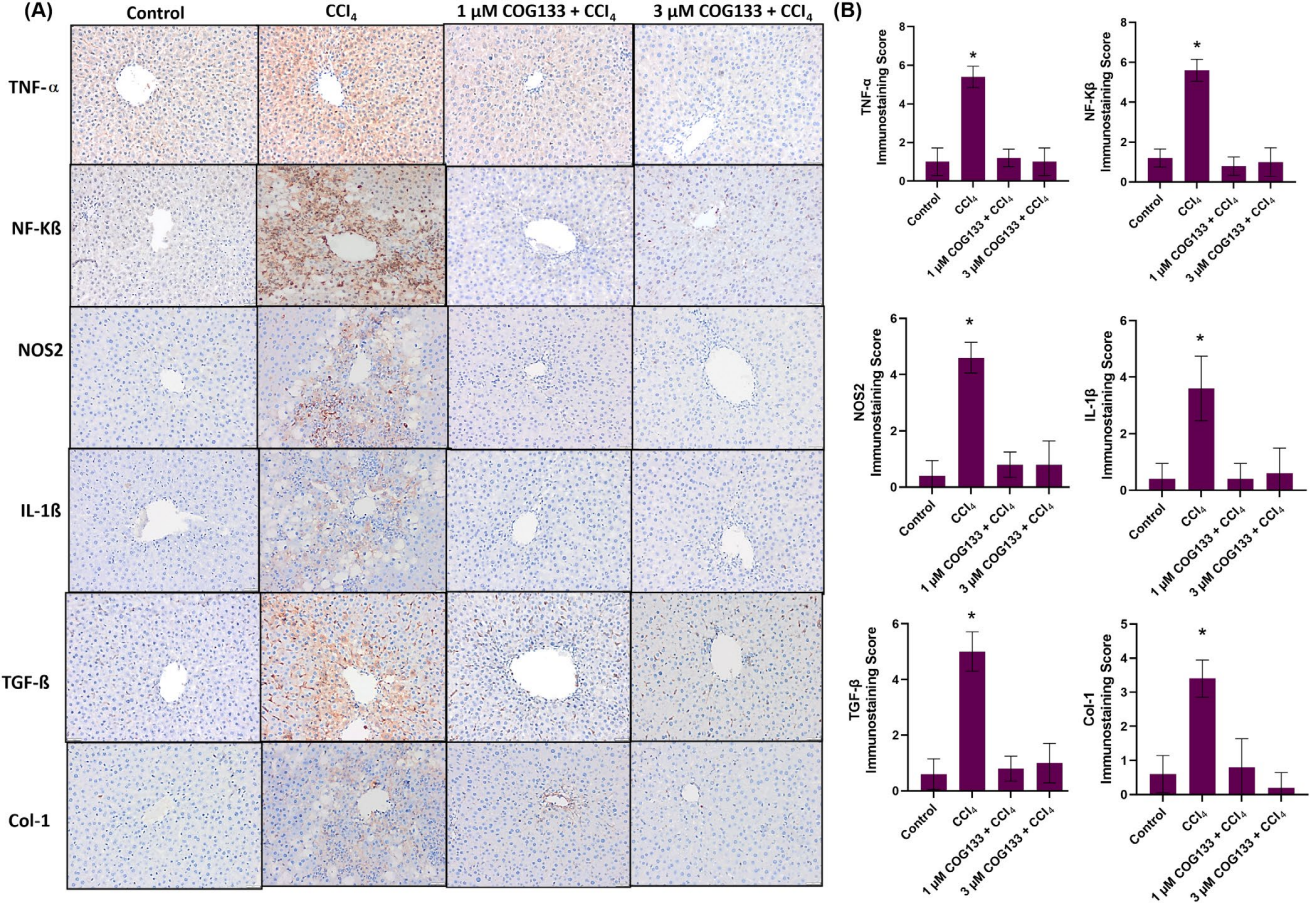
(A) Representative TNF‐α, NF‐KB, NOS2, IL‐1β, TGF‐β, Col‐1 immunoperoxidase staining in liver tissues. Bar, 200 μm. CCl_4_ group, rats treated with CCl_4_ (1 mL/kg body weight/day) for 4 days. 1 μM COG133 + CCl_4_ and 3 μM COG133 + CCl_4_ groups, rats received intraperitoneal COG133 administration of 10 μL/g body weight 2 times a day for 4 days. In these groups, a single dose of CCl_4_ (1 mL/kg body weight/day) was given 1 h after the first COG133 administration. (B) Immunostaining score. Values are given as mean ± SD. *n* = 5. Statistical analysis was conducted by one‐way ANOVA analysis. The difference between the groups was determined by Tukey's test. *p < 0.05, compared to the control and COG133 + CCl_4_ groups.

**FIGURE 3:**
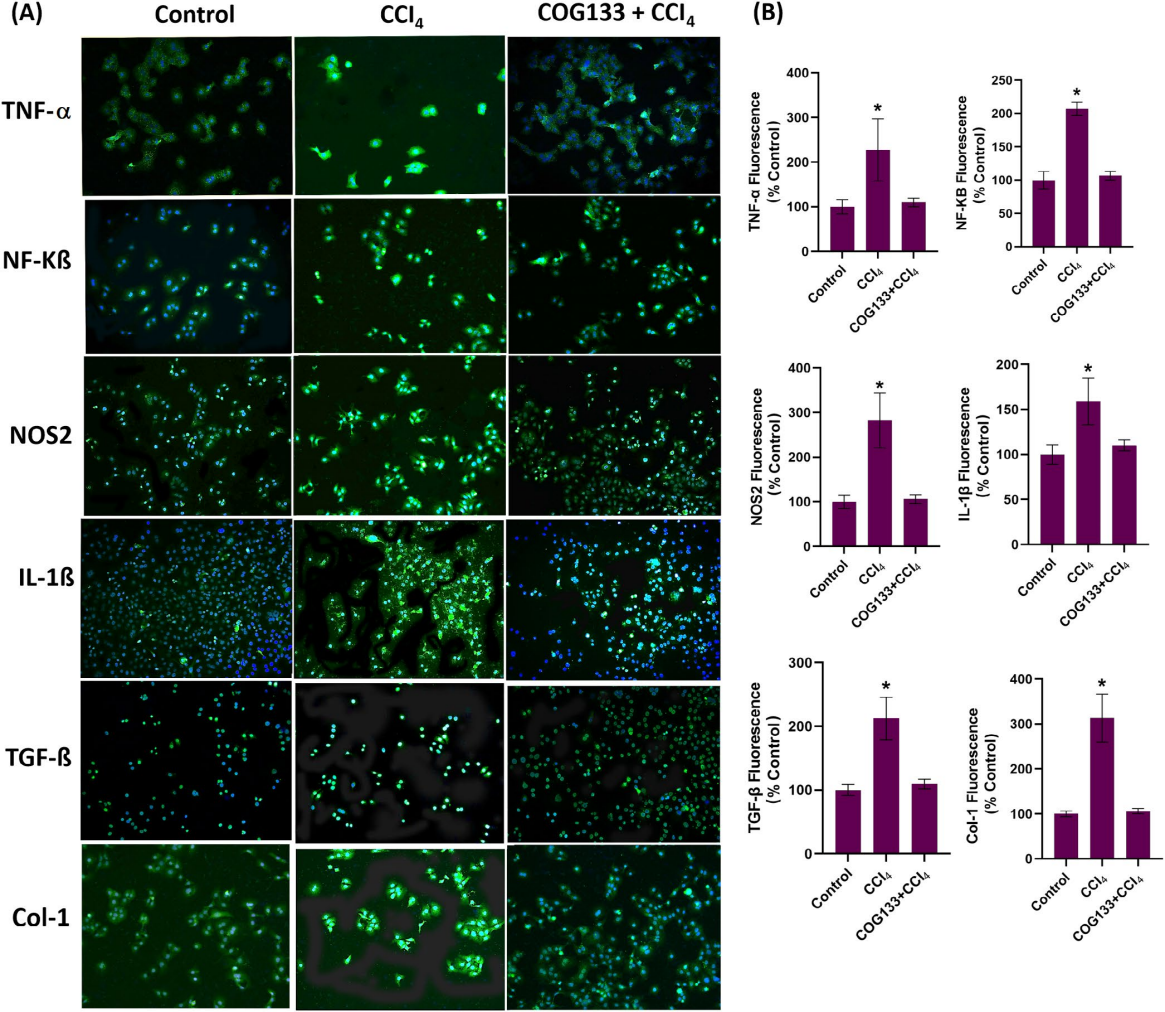
TNF‐α, NF‐KB, NOS2, IL‐1β, TGF‐β and Col‐1 immunofluorescence levels in rat hepatocyte cells. CCl4, cells treated with CCl4 (0.4%) for 6 h; COG133 + CCl_4_, CCl_4_ was administered 18 h after 2 μM COG133 administration (0.4%). The total incubation period is 24 h. (A) Representative immunofluorescence staining images of TNF‐α, NF‐KB, NOS2, IL‐1β, TGF‐β, and Col‐1 in rat hepatocyte cells (10X objective). (B) Quantitation of TNF‐α, NF‐KB, NOS2, IL‐1β, TGF‐β and Col‐1 fluorescent staining by the ImageJ program. The data represents 8 different measurements, and values are mean ± SD. Statistical analysis was conducted by one‐way ANOVA analysis. The difference between groups was determined by Tukey's test. *p < 0.001, compared to all groups.

**FIGURE 4:**
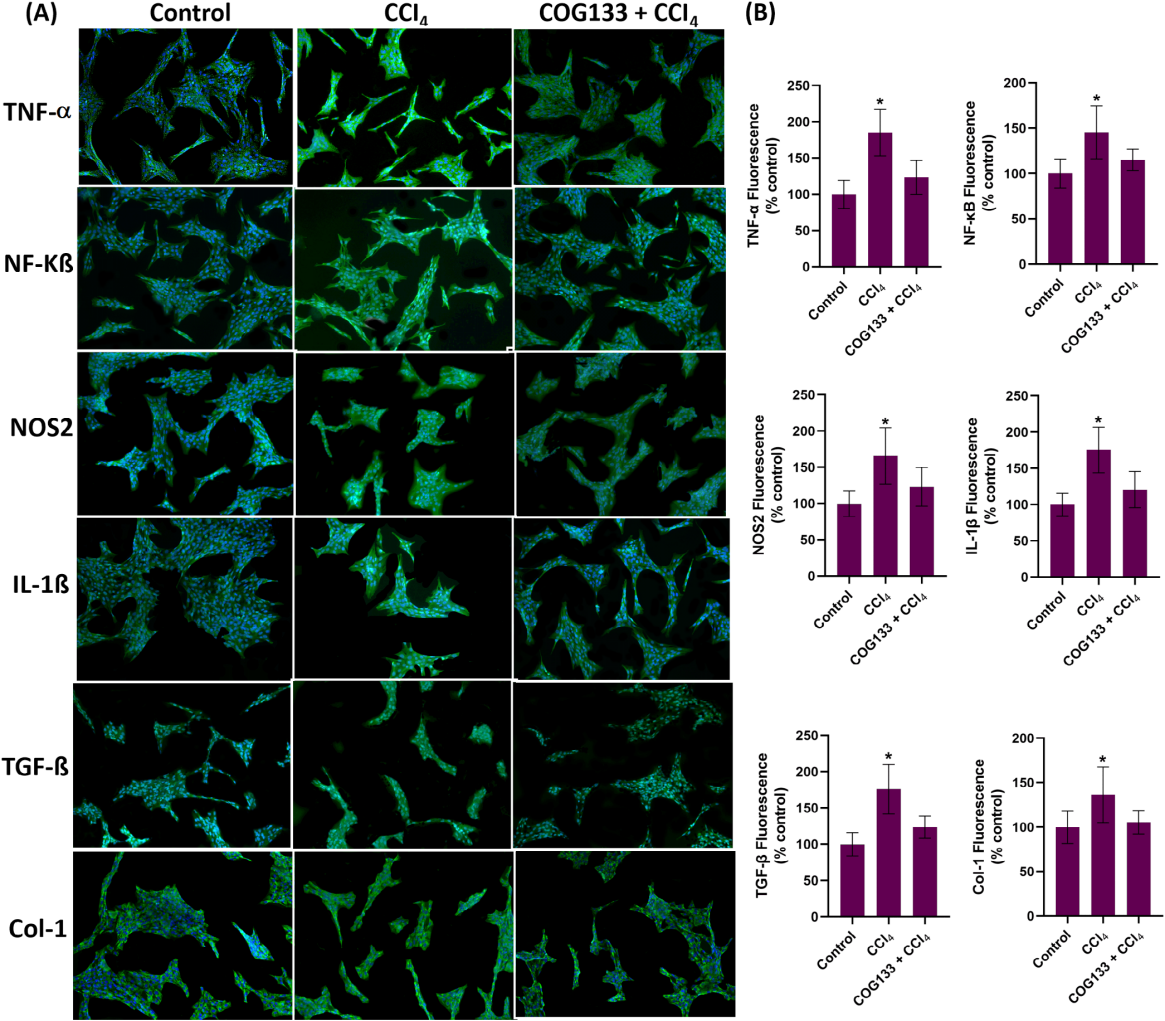
TNF‐α, NF‐KB, NOS2, IL‐1β, TGF‐β, Col‐1 immunofluorescence levels and quantitation in rat hepatic stellate cells. CCl_4_, cells treated with CCl_4_ (0.4%) for 12 h; COG133 + CCl_4_, cells treated with CCl_4_ (0.4%) 12 h after administration of 5 μM COG133. The total incubation period was 24 h. (A) Representative immunofluorescence staining images of TNF‐α, NF‐KB, NOS2, IL‐1β, TGF‐β, Col‐1 in rat hepatic stellate cells. (B) Quantitation of TNF‐α, NF‐KB, NOS2, IL‐1β, TGF‐β, Col‐1 fluorescence staining by the ImageJ program. The data represents 12 separate experiments, and values are mean ± SD. Statistical analysis was conducted by one‐way ANOVA analysis. The difference between groups was determined by Tukey's test. *p < 0.05, compared to the control and COG133 + CCl_4_ groups.

**FIGURE 5:**
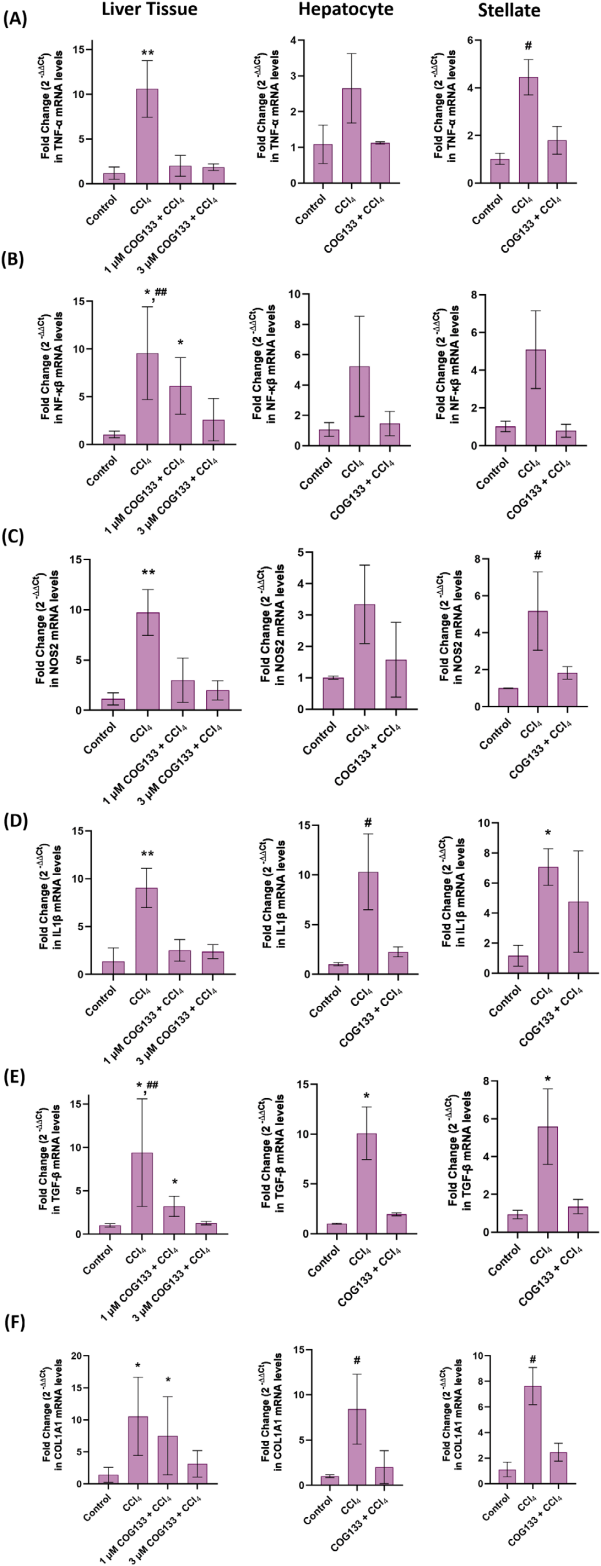
Quantitative PCR analysis in rat liver tissue (*n* = 8), rat hepatocyte cells (*n* = 3), and rat hepatic stellate (*n* = 3) cells. Data shows mean ± SD. Liver CCl_4_ group, rats administered CCl_4_ (1 mL/kg body weight/day) for 4 days. 1 μM COG133 + CCl_4_ and 3 μM COG133 + CCl_4_ groups, rats received intraperitoneal COG133 administration of 10 μL/g body weight 2 times a day for 4 days. In these groups, a single dose of CCl_4_ (1 mL/kg body weight/day) was given 1 h after the first COG133 administration. Hepatocyte CCl_4_ group, cells treated with CCl_4_ (0.4%) for 6 h; Hepatocyte COG133 + CCl_4_ group, cells to which CCl_4_ (0.4%) was administered 18 h after 2 μM COG133 administration. The total incubation time was 24 h. Stellate cell CCl_4_ group, cells treated with CCl_4_ (0.4%) for 12 h; Stellate cell COG133 + CCl_4_ group, CCl_4_ (0.4%) administered 12 h after 5 μM COG133 treatment. The total incubation time was 24 h. Statistical analysis was conducted by one‐way ANOVA analysis or Kruskal‐Wallis test, and the difference between the groups was determined by Tukey's test. *p < 0.05, compared to the control group. **p < 0.05, compared to control, 1 μM COG133 + CCl_4_ and 3 μM COG133 + CCl_4_ groups. ^#^ *p* < 0.05, compared to control and COG133 + CCl_4_ groups. ^##^ *p* < 0.05 compared to the 3 μM COG133 + CCl_4_ group. (A) TNF‐α mRNA levels. (B) NF‐βκ mRNA levels. (C) NOS2 mRNA levels. (D) IL‐1β mRNA levels. (E) TGF‐β mRNA levels. (F) Col‐1 mRNA levels.

To explore the mechanistic basis of COG133's protective effect, markers of inflammation, fibrosis and oxidative stress were examined by immunohistochemistry and immunofluorescence. In liver tissues, CCl_4_ exposure induced a significant increase in the expression of TNF‐α, NF‐κB, NOS2, IL‐1β, TGF‐β and collagen type I (Col‐1) (Figure 2A). This upregulation was effectively suppressed by COG133 treatment, as shown by reduced immunostaining scores (Figure 2B). In vitro, hepatocytes treated with 0.4% CCl_4_ for 6 h exhibited elevated levels of the same inflammatory markers, as evidenced by immunofluorescence staining (Figure 3A). Pre‐treatment with 2 μM COG133 significantly reduced their expression (Figure 3B). In HSC‐T6 cells, a similar pattern was observed following 12 h of CCl_4_ exposure, with 5 μM COG133 suppressing marker expression (Figure 4A,B). Further molecular analyses (Figure 5 and Figure S2) showed that CCl_4_ treatment led to significant upregulation of mRNA and protein levels of these markers, while COG133 administration effectively returned their levels toward baseline in both liver tissues and cultured cells.

### 3.4 Reduction of Apoptosis in Liver Tissue and Cells

**FIGURE 6:**
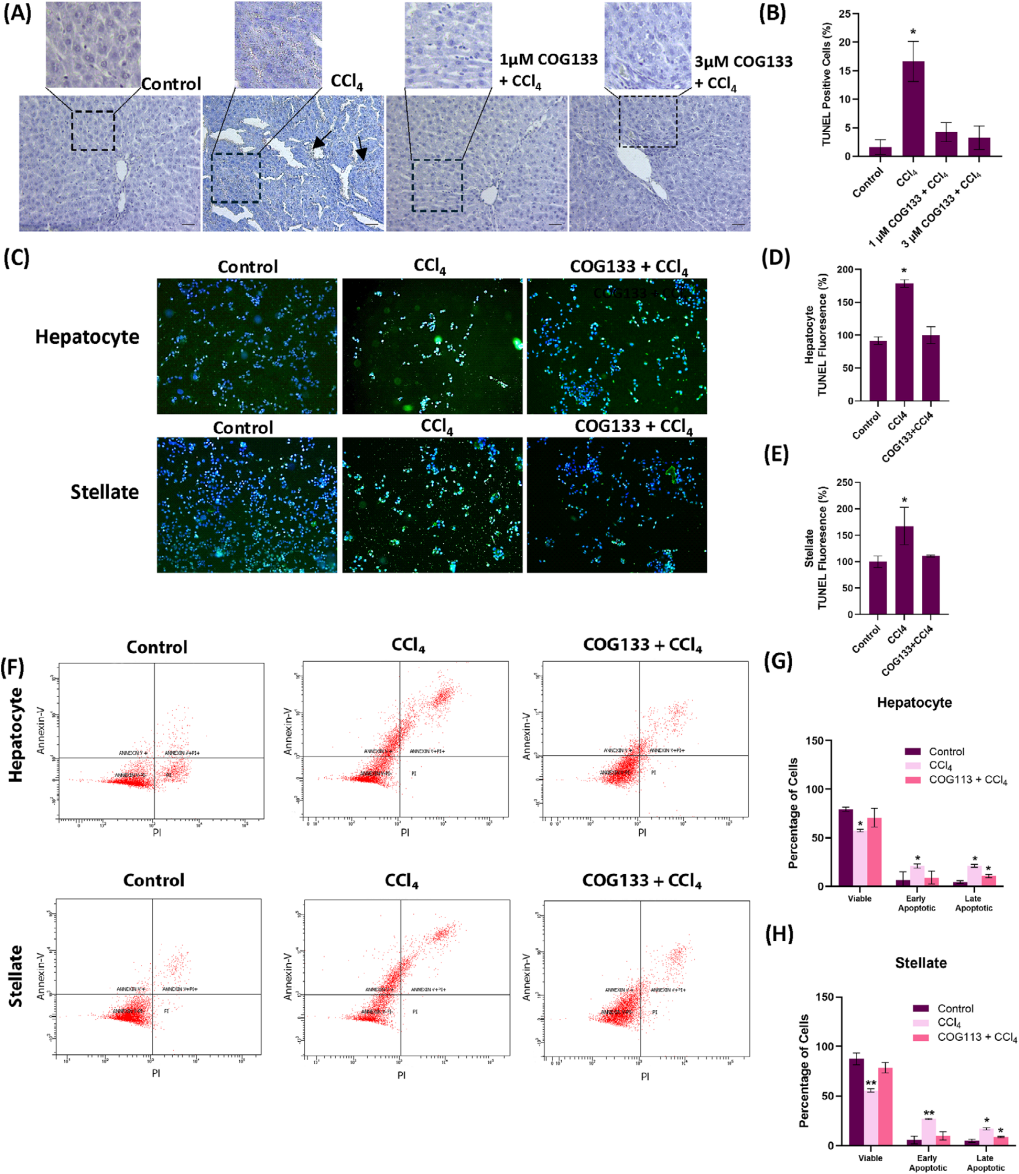
Apoptosis in experimental groups. Liver CCl_4_ group, rats administered a single dose of CCl_4_ (1 mL/kg body weight/day) for 4 days. 1 μM COG133 + CCl_4_ and 3 μM COG133 + CCl_4_ groups, rats received intraperitoneal COG133 administration of 10 μL/g body weight 2 times a day for 4 days. In these groups, a single dose of CCl_4_ (1 mL/kg body weight/day) was given 1 h after the first COG133 administration. Hepatocyte cell CCl_4_ group, cells treated with CCl_4_ (0.4%) for 6 h; Hepatocyte cell COG133 + CCl_4_ group, CCl_4_ (0.4%) was administered 18 h after 2 μM COG133 administration. The total incubation period was 24 h. Stellate cell CCl_4_ group, cells treated with CCl_4_ (0.4%) for 12 h; Stellate cell COG133 + CCl_4_ group, CCl_4_ (0.4%) was administered 12 h after 5 μM COG133 administration. The total incubation period is 24 h. (A) TUNEL staining of liver tissue. Dark brown staining shows TUNEL‐positive cells (arrow) in the CCl_4_ group. Haematoxylin staining was also conducted in which intact cell nuclei were displayed as blue purple. Bar, 200 μm. (B) Quantitation of TUNEL staining with the ImageJ Fiji program. The data represents 8 separate measurements, and the values are given as mean ± SD. Statistical analysis was conducted by one‐way ANOVA test and the difference between groups was determined by Tukey multiple comparison test. *p < 0.001, compared to all groups. (C) Immunofluorescent TUNEL staining in rat hepatocyte and stellate cells. (D, E) Quantitation of TUNEL staining in rat hepatocyte and stellate cells. The data represents 8 separate measurements, and values are mean ± SD. Statistical analysis was conducted by one‐way ANOVA analysis. The difference between the groups was determined by Tukey's test. *p < 0.05, compared to all experimental groups. (F) Representative flow cytometry analysis of Annexin V–FITC and PI‐labelled rat hepatocyte and hepatic stellate cells. In each panel, the lower left quadrant shows viable cells, the upper left quadrant shows early apoptotic cells, the upper right quadrant shows late apoptotic cells, and the lower right quadrant shows necrotic cells. (G) Quantitative analysis of Annexin‐V and PI labeling in rat hepatocyte cells. Data represents three separate experiments and values are given as mean ± SD. Statistical analysis was conducted by one‐way ANOVA analysis. The difference between groups was determined by Tukey test. *p < 0.05, compared to control group. (H) Quantitative analysis of Annexin‐V and PI labeling in rat hepatic stellate cells. Data represents three separate experiments and values are given as mean ± SD. Statistical analysis was conducted by one‐way ANOVA analysis. The difference between groups was determined by Tukey test. *p < 0.05, compared to control group. **p < 0.05, compared to control and COG133 + CCl_4_ groups.

The anti‐apoptotic effect of COG133 was evaluated using TUNEL staining and flow cytometry. In liver tissue, TUNEL‐positive cells were markedly increased following CCl_4_ administration (Figure 6A), with quantification revealing significant differences compared to controls (Figure 6B). COG133 treatment significantly reduced TUNEL‐positive cells. Similarly, TUNEL staining of hepatocytes and stellate cells showed that CCl_4_ induced marked apoptosis, which was attenuated by COG133 treatment (Figure 6C). Quantitative analyses confirmed that COG133 reduced apoptotic cell percentages in both cell types (Figure 6D,E). Flow cytometry analyses further corroborated these findings, showing increased early and late apoptotic populations after CCl_4_ exposure (Figure 6F). COG133 significantly decreased both early and late apoptosis rates in hepatocytes and HSC‐T6 cells (Figure 6G,H).

### 3.5 Modulation of Sphingolipid Metabolism

Sphingolipid analyses revealed that levels of sphingomyelins (16:0 SM, 18:0 SM and 24:0 SM) and S1P were significantly reduced in liver tissue, hepatocytes and HSC‐T6 cells following CCl_4_ exposure (Table 1). Treatment with COG133 partially restored SM and S1P levels in liver tissues, although full normalisation was not achieved. In hepatocytes and stellate cells, while COG133 improved S1P levels, SM levels (16:0–24:0 SM) remained significantly lower compared to control groups. Moreover, ceramide species (C16–C24 CERs) were reduced following CCl_4_ treatment, and COG133 did not significantly restore these levels. These findings suggest that while COG133 provides partial correction of sphingolipid dysregulation, ceramide metabolism may require alternative or additional therapeutic approaches.

**TABLE 1:** Sphingolipid levels.

	Sphingomyelin (ng/mg protein)				Ceramide (ng/mg protein)					
	16:0 SM (d18:1/16:0)	18:0 SM (d18:1/18:0)	24:0 SM (d18:1/24:0)	S1P	C16 CER (d18:1/16:0)	C16 CER‐1P (d18:1/16:0)	C18 CER (d18:1/18:0)	C20 CER (d18:1/20:0)	C22 CER (d18:1/22:0)	C24 CER (d18:1/24:0)
Liver tissue										
Control	227.39 ± 87.78	70.74 ± 20.11	177.84 ± 97.22	5.36 ± 1.27	175.44 ± 46.14	9.20 ± 1.47	19.91 ± 3.72	15.75 ± 6.57	51.79 ± 11.23	218.93 ± 53.15
CCI_4_	70.84 ± 21.10^a^	16.46 ± 5.44^d^	58.50 ± 19.35^d^	2.50 ± 0.96^e^	51.53 ± 8.06^g^	3.06 ± 1.57^e^	5.97 ± 0.91^h^	4.22 ± 0.48^a,d^	16.45 ± 3.76^h^	56.44 ± 15.61^g^
COG133 + CCI_4_ (1 μM)	121.15 ± 24.06	34.34 ± 8.53	110.73 ± 37.92	4.85 ± 2.64	84.30 ± 19.02	3.90 ± 2.14^e^	10.45 ± 2.92^h,j^	10.68 ± 2.29	25.75 ± 8.56^h^	86.12 ± 26.16^e^
COG133 + CCI_4_ (3 μM)	145.04 ± 51.85	31.48 ± 12.04^e^	95.73 ± 50.29	4.08 ± 0.99	64.09 ± 15.45^e^	3.56 ± 2.67^e^	11.85 ± 2.09^h,j^	12.42 ± 3.84	21.56 ± 5.87^h^	86.67 ± 23.32^e^
Hepatocyte										
Control	174.94 ± 10.02	51.69 ± 9.16	14.52 ± 1.00	5.25 ± 1.21	70.31 ± 25.78	5.72 ± 2.00	52.36 ± 17.13	7.20 ± 1.17	30.54 ± 3.25	57.55 ± 20.72
CCI_4_	42.18 ± 2.60^b^	12.48 ± 1.83^f^	3.15 ± 0.71^b^	2.34 ± 0.73^h^	6.70 ± 1.92^g^	0.83 ± 0.40^g^	7.02 ± 2.96^e^	2.64 ± 0.46^b^	5.45 ± 1.22^b^	16.40 ± 7.21^e^
COG133 + CCI_4_	89.15 ± 3.19^c^	36.99 ± 12.43	6.71 ± 1.22^c^	3.31 ± 0.70^i^	12.29 ± 1.33	1.28 ± 0.25	9.65 ± 1.31^e^	5.36 ± 1.01^c^	10.39 ± 1.02^c^	19.42 ± 1.60^e^
Stellate										
Control	201.35 ± 27.76	27.56 ± 1.50	9.42 ± 0.81	3.51 ± 1.10	9.63 ± 0.57	2.74 ± 0.54	5.63 ± 0.42	3.19 ± 0.28	6.58 ± 0.45	22.33 ± 3.71
CCI_4_	37.40 ± 7.36^b^	5.80 ± 1.45^g^	2.68 ± 0.17^b^	2.96 ± 1.72	3.26 ± 0.51^g^	1.14 ± 0.74^e^	2.80 ± 0.45^b^	2.28 ± 0.65^e^	3.84 ± 1.20^g^	10.20 ± 2.95^h^
COG133 + CCI_4_	148.31 ± 18.11^c^	17.20 ± 3.83	7.66 ± 0.72^c^	3.32 ± 0.63	5.29 ± 1.53	1.22 ± 0.67^e^	4.09 ± 0.58^c^	2.71 ± 0.39	5.20 ± 0.41	16.89 ± 2.58^j,i^

*Note:* All values are given as mean ± SD. *n* = 8 in liver tissue and *n* = 6 in hepatocyte and stellate cell lysates. N‐palmitoyl‐d‐erythro‐sphingosylphosphorylcholine (C16 SM), N‐stearoyl‐d‐erythro‐sphingosylphosphorylcholine (C18 SM), N‐lignoceroyl‐d‐erythro‐sphingosylphosphorylcholine (C24 SM), N‐palmitoyl‐d‐erythro‐sphingosine (C16 CER), N‐stearoyl‐d‐erythro‐sphingosine (C18 CER), N‐arachidoyl‐d‐erythro‐sphingosine (C20 CER), N‐behenoyl‐d‐erythro‐sphingosine (C22 CER), N‐lignoceroyl‐d‐erythro‐sphingosine (C24 CER), d‐erythro‐sphingosine‐1‐phosphate (S1P), and N‐palmitoyl‐CER‐1‐phosphate (C16 C1P). ^a^ *p* < 0.05 vs. control and 3 μM COG133 + CCI4 (Kruskal‐Wallis One Way Analysis of Variance on Ranks and All Pairwise Multiple Comparison Procedures via Tukey Test). ^b^ *p* < 0.001 vs. control and COG133 + CCI4 (One Way Analysis of Variance and All Pairwise Multiple Comparison Procedures via Tukey Test). ^c^ *p* < 0.01 vs. control and CCI4 (One Way Analysis of Variance and All Pairwise Multiple Comparison Procedures via Tukey Test). ^d^ *p* < 0.05 vs. control and 1 μM COG133 + CCI4 (Kruskal‐Wallis One Way Analysis of Variance on Ranks and All Pairwise Multiple Comparison Procedures via Tukey Test). ^e^ *p* < 0,05 vs. control (Kruskal‐Wallis One Way Analysis of Variance on Ranks and All Pairwise Multiple Comparison Procedures via Tukey Test). ^f^ *p* < 0,05 vs. control and COG133 + CCI4 (Kruskal‐Wallis One Way Analysis of Variance on Ranks and All Pairwise Multiple Comparison Procedures via Tukey Test). ^g^ *p* < 0,001 vs. control (Kruskal‐Wallis One Way Analysis of Variance on Ranks and All Pairwise Multiple Comparison Procedures via Tukey Test). ^h^ *p* < 0.001 vs. control (One Way Analysis of Variance and All Pairwise Multiple Comparison Procedures via Tukey Test). ^i^ *p* < 0.05 vs. control (One Way Analysis of Variance and All Pairwise Multiple Comparison Procedures via Tukey Test). ^j^ *p* < 0.01 vs. CCI4 (One Way Analysis of Variance and All Pairwise Multiple Comparison Procedures via Tukey Test).

## 4 Discussion

The focus on COG133 as a potential therapeutic agent for liver injury ties directly to translational medicine. Although the study used rat models and hepatic cell lines, the findings provide insights into potential human applications. The rat liver shares many structural and functional similarities with the human liver, including metabolic pathways involving cytochrome P450 enzymes, essential in the biotransformation of xenobiotics. CCl_4_ is primarily used in refrigerant production and has other industrial applications, such as fire extinguishers, dry‐cleaning agents and pesticide dispersants [27]. Though banned in household products, it remains used in polymer manufacturing and aerosol propellants. After absorption, CCl_4_ distributes to organs, peaking in 1–6 h, with buildup in adipose tissue [28]. CCl_4_ is commonly used in experimental research to model liver injury. It is metabolised in the liver primarily by the cytochrome P450 enzyme system, particularly CYP2E1 and CYP2B1, producing highly reactive trichloromethyl radicals (∙O‐O‐CCl_3_) that initiate lipid peroxidation, react with proteins and disrupt cellular structures, leading to cell death [4]. Our study administered CCl_4_ in rats via subcutaneous injection to induce acute liver hepatotoxicity. Administration of CCl_4_ resulted in marked cloudy swelling, steatosis and necro‐inflammatory activity around the central vein, consistent with previous studies showing centrilobular necrosis due to high CYP2E1 expression in this region [29, 30]. CCl_4_ treatment did not cause significant changes in body weight or liver‐to‐body weight ratio, indicating that liver compensatory mechanisms, like regenerative proliferation, might maintain liver mass despite cellular damage [31]. The absence of notable fibrosis or steatosis could also contribute to the maintenance of liver mass and thus not impact the liver‐to‐body weight ratio significantly [32]. Short‐term exposure to CCl_4_ has been reported to elevate liver enzymes without significantly altering liver size [33].

Presented data show dose‐ and time‐dependent effects of COG133 on cell viability and its protective efficacy against CCl_4_‐induced toxicity in both rat hepatocytes and HSC‐T6 cells. The data confirm CCl_4_'s known cytotoxicity [34, 35]. In hepatocytes, toxic effects appeared after 3 h of 0.4% CCl_4_ exposure, with severe viability reduction after 6 h, in line with previous reports [36]. In HSC‐T6 cells, significant toxicity emerged after 12 h of CCl_4_ exposure, with viability declining sharply at 24 h [37]. Administration of COG133 significantly enhanced cell viability in both hepatocytes and HSC‐T6 cells, with the optimal dose for hepatocytes being 2 μM and for HSC‐T6 cells, 2–10 μM. These findings suggest that COG133 provides cytoprotection across both cell types, highlighting its broad protective potential. Morphological analysis corroborated the viability data, with CCl_4_ exposure inducing typical signs of cellular distress, such as shrinkage, detachment and rounding. COG133 treatment alleviated these disruptions, preserving cellular integrity and preventing oxidative and structural damage. Additionally, COG133 has shown promise in protecting intestinal cells from chemotherapy‐induced damage by reducing apoptosis and enhancing cell migration [17]. Studies have indicated that ApoE (133–150), a segment encompassing COG133, is non‐toxic to several human cell lines and can trigger a significant innate immune response, further supporting its protective role in cellular contexts [38]. These results position COG133 as a promising candidate for protecting liver cells against chemical‐induced injury. Its efficacy in both primary hepatocytes and HSC‐T6 cells indicates its potential utility in diverse hepatic cell types, including parenchymal and stellate cells. The dose‐ and time‐dependent effects underscore the importance of optimising treatment parameters for maximal benefit.

Upon exposure, CCl_4_ induces hepatocyte damage, resulting in the formation of damage‐associated molecular patterns [39], which activate Kupffer cells, triggering an inflammatory response that further aggravates liver injury [40]. Pro‐inflammatory cytokines like TNF‐α and IL‐6 promote inflammation, and repeated exposure to CCl_4_ stimulates HSCs, leading to collagen deposition and fibrosis [41, 42]. TGF‐β plays a key role in liver fibrosis by activating HSCs, which then produce excessive extracellular matrix components, including collagen [43]. Studies suggest that inhibiting TGF‐β signalling can reduce fibrogenesis [44]. In this study, COG133 treatment significantly reduced pro‐inflammatory and fibrotic markers, including TNF‐α, NF‐κB, NOS2, IL‐1β, TGF‐β and Col‐1, aligning with studies demonstrating that ApoE‐mimetic peptides suppress inflammatory cascades. For instance, ApoE [133–149] peptide therapy was shown to inhibit microglial activation and reduce pro‐inflammatory mediators in models of neuroinflammation [15].

ApoE‐mimetic peptides have been shown to reduce inflammation in models of atherosclerosis. These peptides dramatically reduced atherosclerotic lesions in animal models, highlighting their potential therapeutic role in inflammatory cardiovascular diseases [45]. The reduction in TNF‐α and NF‐κB levels mirrors studies where COG133 or similar peptides reduced NF‐κB activation, mitigating inflammation and cell death. For instance, COG112, a fusion peptide enhancing the bioactivity of COG133, inhibited NF‐κB signalling and reduced proinflammatory cytokine expression in murine models of colitis, indicating its potential to suppress inflammatory pathways in gastrointestinal inflammation [46]. COG133 also reduced the levels of inducible NOS2, which is involved in oxidative stress during CCl_4_‐induced liver injury [47]. ApoE‐mimetic peptides have been shown to attenuate NOS2 expression in models of ischemia–reperfusion injury, thus preserving tissue integrity [48]. COG133's inhibition of TGF‐β and Col‐1 expression suggests its potential in preventing fibrosis, as it reduced collagen production in both hepatocytes and stellate cells [49]. Furthermore, the role of ApoE in modulating fibroblast activity and resolving fibrosis has been demonstrated in pulmonary fibrosis models, where ApoE promoted the resolution of fibrosis by binding to collagen and mediating its phagocytosis [50].

COG133 significantly reduced TUNEL‐positive cells in liver tissue, hepatocytes and stellate cells, suggesting that it mitigates CCl_4_‐induced apoptosis by counteracting oxidative stress or modulating apoptotic signalling pathways. Stellate cells, which are crucial in fibrosis, are protected from apoptosis, which may help prevent fibrosis progression. The flow cytometry results, based on annexin V/PI staining, quantified early and late apoptotic cells, confirming the TUNEL assay's findings [51].

Additionally, we investigated serum sphingolipid levels and their modulation by COG133 treatment. CCl_4_ exposure led to a decrease in SMs and S1P, which are crucial for cell membrane integrity, signalling and apoptosis regulation. This is consistent with prior research, which has shown that oxidative stress and liver injury induced by CCl_4_ can disrupt sphingolipid metabolism [52]. S1P has been shown to protect intestinal epithelial cells from apoptosis via an Akt‐dependent pathway, highlighting its role in cell survival [53]. Sphingomyelin hydrolysis leads to CER generation, which in turn activates apoptotic pathways. The loss of plasma membrane lipid asymmetry, including the depletion of SM, is a significant event during apoptosis [54]. The depletion of these sphingolipids suggests disrupted cell survival pathways. While COG133 partially restored S1P levels, it did not significantly restore CER levels, indicating that COG133 might influence S1P signalling but not directly affect CER production. This partial restoration suggests a therapeutic potential in modulating sphingolipid metabolism, which is implicated in liver diseases like NASH and fibrosis [55, 56].

In conclusion, the results provide compelling evidence that COG133 can attenuate inflammation and fibrosis in liver injury, suggesting its potential as a therapeutic agent for inflammatory and fibrotic diseases. COG133's ability to enhance cell viability, protect against morphological disruptions and provide broad cytoprotection across cell types highlights its promise in liver injury prevention and therapy. Further studies are needed to explore its mechanisms of action, long‐term safety and clinical relevance. Although promising, the study also has limitations. Utilising CCl_4_ as the sole model may not capture all drug‐induced liver injury mechanisms. Conducted in rats, species differences limit generalizability. Long‐term effects and interactions with standard treatments remain unexplored. Future studies should include other models and explore COG133's potential in combination therapies. Another limitation of this study is the evaluation of only two COG133 doses, which were chosen based on previous efficacy studies in murine models. While this provides a starting point, a broader dose–response analysis would be valuable for optimising therapeutic dosing. Additionally, the use of rat hepatocytes and hepatic stellate cell lines, while well‐established and informative, may not fully recapitulate the complexity of human liver physiology. Future investigations should include a wider range of doses and human liver models to enhance translational applicability.

## Author Contributions

**Mutay Aslan:** conceptualization (lead), formal analysis (supporting), funding acquisition (lead), investigation (supporting), methodology (lead), project administration (lead), supervision (lead), writing – original draft (lead), writing – review and editing (lead). **Bürke Çırçırlı:** formal analysis (equal), investigation (equal), methodology (equal), project administration (equal). **Aleyna Öztüzün:** formal analysis (equal), investigation (equal), methodology (equal), project administration (equal). **Hazal Tuzcu:** formal analysis (equal), investigation (equal), methodology (equal). **Çağatay Yılmaz:** formal analysis (equal), investigation (equal), methodology (equal). **Tuğçe Çeker:** formal analysis (equal), investigation (equal), methodology (equal). **Gülsüm Özlem Elpek:** formal analysis (equal), investigation (equal), methodology (equal).

## Ethics Statement

This study was reviewed and approved by Akdeniz University Experimental Animals Application and Research Center, Animal Experiments Local Ethics Committee (Decision No: 30‐Date: 09.03.2023).

## Conflicts of Interest

The authors declare no conflicts of interest.

## Supporting information


Figure S1.

Figure S2.

Table S1.

Table S2.

## Data Availability

Data obtained and analysed are available from the corresponding author on reasonable request.
