# Supplementary material for: Protective Effects of COG133 on Carbon Tetrachloride‐Induced Acute Liver Injury: Modulation of Inflammation, Apoptosis and Sphingolipid Metabolism
Source: J Cell Mol Med. 2025 Jun 21;29(12):e70677. doi: 10.1111/jcmm.70677 (PMC12181747; doi:10.1111/jcmm.70677)
Supplement: Supplementary file 1 — Figure S1. [file JCMM-29-e70677-s001.pdf]

Supplementary Figure 1

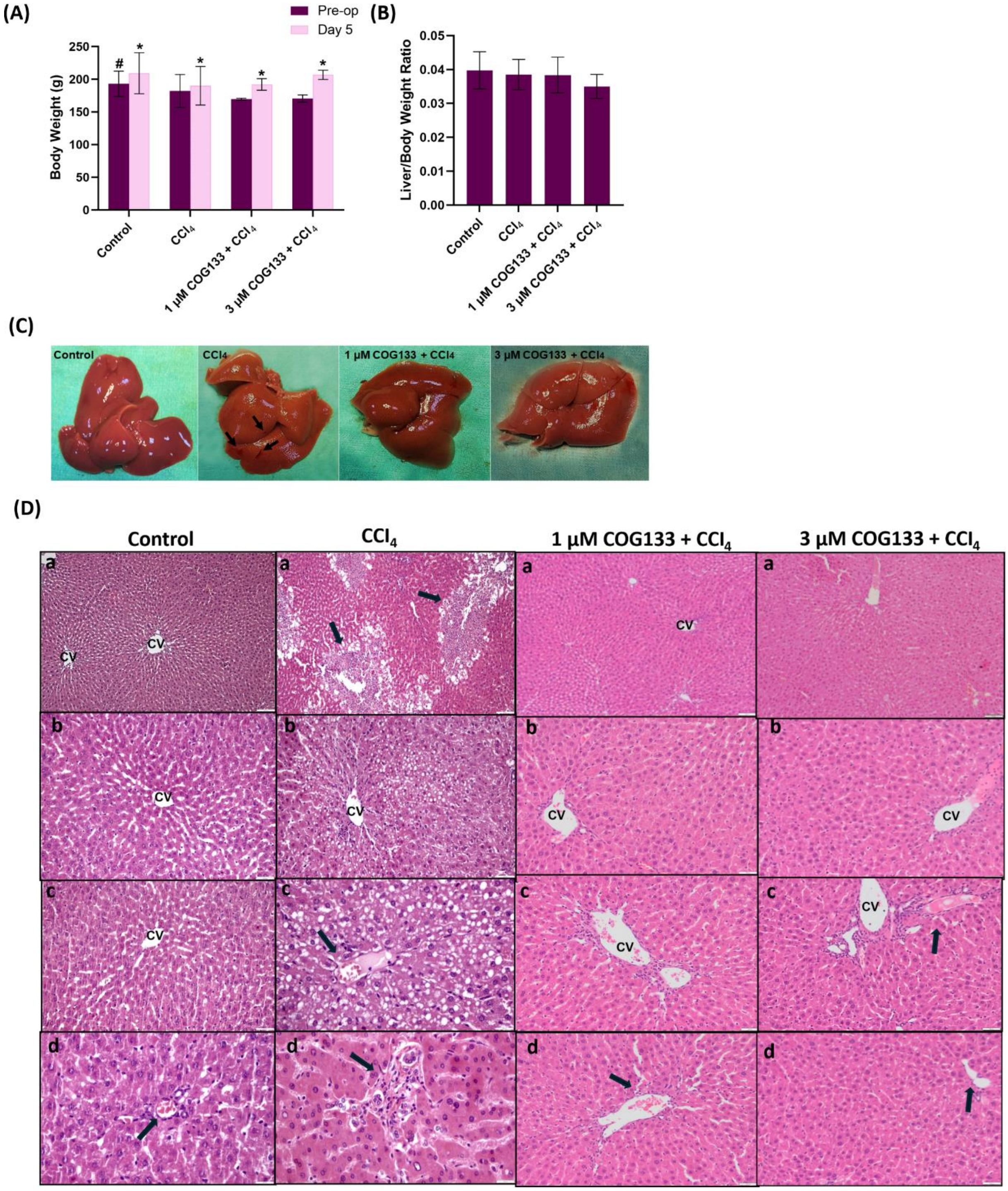

Figure 1. Changes in liver and body weight. **(A)** Comparison of body weights before and after acute hepatotoxicity. Data are from 8 rats, values are mean ± SD. Statistical analysis was with one-way ANOVA analysis between groups followed by Tukey's multiple comparison test. Statistical analysis within the same group was by paired-t test. *, $p<0.05$, compared with pre-op within the same group. #, $p<0.05$ compared to 1 μM COG133 + CCl_4 and 3 μM COG133 + CCl_4 groups. **(B)** Ratio of liver weight to body weight. Data represents 8 rats, and values are

mean ± SD. **(C)** Representative photographs of the liver from experimental groups. **(D)** Representative microphotographs of H&E staining in liver tissues removed 24 hours after CCl_4 administration. Liver CCl_4 group, rats administered a single dose of CCl_4 (1 ml/kg body weight/day) for 4 days. 1 µM COG133 + CCl_4 and 3 µM COG133 + CCl_4 groups, rats received intraperitoneal COG133 administration of 10 µl/gram body weight 2 times a day for 4 days. In these groups, a single dose of CCl_4 (1 ml/kg body weight/day) was given 1 hour after the first COG133 administration. Bar, 200 µm. CV, central vein. Control (a), (b) and (c) show the general microscopic view of liver tissue. Control (d) shows the portal triad (arrow) consisting of the portal vein, arteriole and interlobular bile duct. CCl_4 (a) shows confluent necrosis (arrow). CCl_4 (b) shows lytic necrosis and adiposity. CCl_4 (c) shows widespread adiposity and cell loss (arrow). CCl_4 (d) shows portal inflammation and interface hepatitis (arrow). 1 µM and 3 µM COG133 + CCl_4 (a), (b) and (c) shows microscopic view of liver tissue. There is a mild degree of combined necrosis caused by inflammatory cells that cause hepatocyte loss in the lobules. Focal lytic necrosis within the lobule is also seen (arrow). 1 µM and 3 µM COG133 + CCl_4 (d) shows interface hepatitis formed by lymphocytes surrounding hepatocytes in the portal space and disrupting the limiting plaque, as well as moderate inflammatory infiltration of lymphocytes (arrow).
