# Supplementary material for: Protective Effects of COG133 on Carbon Tetrachloride‐Induced Acute Liver Injury: Modulation of Inflammation, Apoptosis and Sphingolipid Metabolism
Source: J Cell Mol Med. 2025 Jun 21;29(12):e70677. doi: 10.1111/jcmm.70677 (PMC12181747; doi:10.1111/jcmm.70677)
Supplement: Supplementary file 2 — Figure S2. [file JCMM-29-e70677-s004.pdf]

Supplementary Figure 2

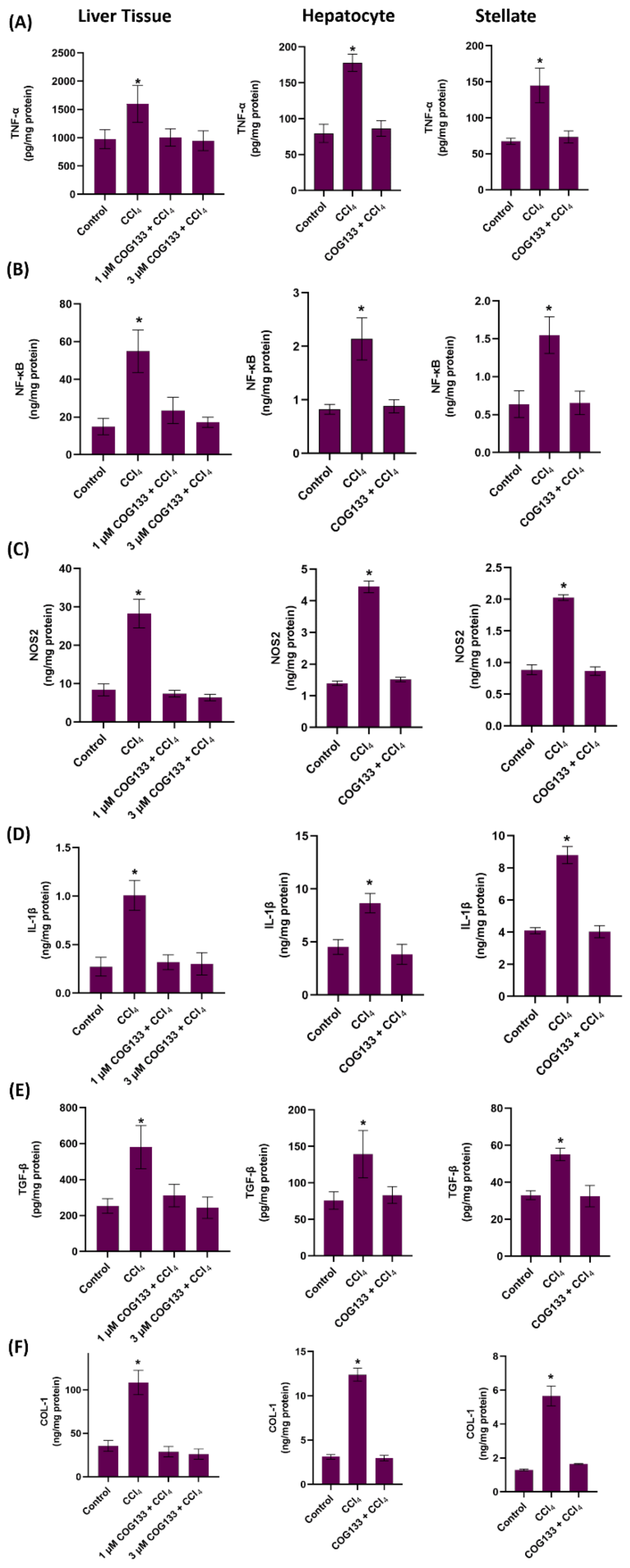

Supplementary Figure 2. ELISA results in rat liver tissue (n=8), rat hepatocyte cells (n=6) and rat hepatic stellate (n=6) cells. Data shows mean ± SD. **(A)** TNF- α levels. Statistical analysis was performed with the Kruskal-Wallis test and difference between groups was determined by Dunn's multiple comparison test. *, $p<0.05$, CCl_4 group compared to other experimental groups. **(B)** NF-κβ levels. Statistical analysis was performed by one-way ANOVA analysis and difference between groups was determined by Tukey test. *, $p<0.001$, CCl_4 group compared with other experimental groups. **(C)** NOS2 levels. Statistical analysis was performed with one-way ANOVA test and difference between groups was determined by Tukey multiple comparison test. *, $p<0.001$, CCl_4 group compared with other experimental groups. **(D)** IL-1β levels. Statistical analysis was performed by one-way ANOVA test and difference between groups was determined by Tukey multiple comparison test. *, $p<0.001$, CCl_4 group compared with other experimental groups**. (E)** TGF-β levels. Statistical analysis was performed by one-way ANOVA test and difference between the groups was determined by Tukey multiple comparison test. *, $p<0.05$, CCl_4 group compared with other experimental groups. **(F)** Col-1 levels. Statistical analysis was performed with one-way ANOVA test and difference between groups was determined by Tukey multiple comparison test. *, $p<0.001$, CCl_4 group compared with other experimental groups.
