# Supplementary material for: Protective Effects of COG133 on Carbon Tetrachloride‐Induced Acute Liver Injury: Modulation of Inflammation, Apoptosis and Sphingolipid Metabolism
Source: J Cell Mol Med. 2025 Jun 21;29(12):e70677. doi: 10.1111/jcmm.70677 (PMC12181747; doi:10.1111/jcmm.70677)
Supplement: Supplementary file 3 — Table S1. [file JCMM-29-e70677-s002.pdf]

Supplementary Table 1

GENE	OLIGO NAME	SEQUENCE	5' DYE	3'-DYE	GENBANK / NO	BRAND	ORIGIN
**COL1A1**	COL1A1P1	GGATGGCTGCACGAGTCACAC	-	-	RGD/61817	METABION	GERMANY
	COL1A1P2	CAGATTGGGATGGAGGGAGTTTAC	-	-		METABION	GERMANY
	COL1A1PR	ACTTGGGGCAAGACAGTCATCGAATACAA	FAM	BHQ-1		METABION	GERMANY
**IL1B**	Il1bP1	GCTTCAAATCTCACAGCAGCATCT	-	-	RGD/2891	METABION	GERMANY
	Il1bP2	ACAGAGGACGGGCTCTTCTTC	-	-		METABION	GERMANY
	Il1bPR	AGAGCTTCAGGAAGGCAGTGTCACTC	FAM	BHQ-1		METABION	GERMANY
**NFKB**	ReLaP1	CCAACACTGCCGAGCTCAAGAT	-	-	RGD/727889	METABION	GERMANY
	ReLaP2	ACACCTCAATGTCTTCTTTCTGCAC	-	-		METABION	GERMANY
	ReLaPR	CGAGTAAACCGGAACTCTGGGAGCT	FAM	BHQ-1		METABION	GERMANY
**Rn18s**	Rat18srnaP1	CGGACAGGATTGACAGATTGATAG	-	-	RGD/5687330	METABION	GERMANY
	Rat18srnaP2	GTCTCGTTCGTTATCGGAATTAAC	-	-		METABION	GERMANY
	Rat18srnaPR	CTCGATTCCGTGGGTGGTGGTGC	FAM	BHQ-1		METABION	GERMANY
**NOS2**	NOSRATP1	TGGCCTCCCTCTGGAAAGAC	-	-	RGD/3185	SFC	SOUTH KOREA
	NOSRATP2	GGTGGTCCATGATGGTCACATTC	-	-		SFC	SOUTH KOREA
	MUNOSPR	TCACCGAGATCAATGCAGCTGTGCTC	FAM	BHQ-1		PROBSNYTESIS	TÜRKİYE
**TNF**	TNFYP1	GGCCTCCAGAACTCCAGGCGGTGT	-	-	RGD/3876	PROBSYNTESIS	TÜRKİYE
	TNFRATKP2	GACTCCGTGATGTCTAAGTACTTG	-	-		SFC	SOUTH KOREA
	TNFPR	TGGGCTCCCTCTCATCAGTTCCATG	FAM	BHQ-1		METABION	ALMANYA
**TGFB**	TGFB1RATP1	TGACCTGGGCACCATCCATGA	-	-	RGD/69051	SFC	SOUTH KOREA
	TGFB1RATP2	GTCAATGTACAGCTGCCGTACAC	-	-		SFC	SOUTH KOREA
	TGFBYPR	ACCGACCCTTCCTGCTCCTCATG	FAM	BHQ-1		PROBSYNTESIS	TÜRKİYE

Supplementary Table 1. List of oligonucleotide sequences used for gene expression analysis. The table includes gene names, corresponding primer and probe identifiers, nucleotide sequences, dye labels at the 5′ and 3′ ends, GenBank reference numbers, and the suppliers/origins of the oligonucleotides. Fluorescent probes are labelled with 5-FAM (5-Carboxyfluorescein) at the 5′ end and BHQ-1 (Black Hole Quencher-1) at the 3′ end. All primers and probes were synthesized by Metabion (Germany), SFC (South Korea), or ProbsynTesis (Türkiye).
