# Supplementary material for: Protective Effects of COG133 on Carbon Tetrachloride‐Induced Acute Liver Injury: Modulation of Inflammation, Apoptosis and Sphingolipid Metabolism
Source: J Cell Mol Med. 2025 Jun 21;29(12):e70677. doi: 10.1111/jcmm.70677 (PMC12181747; doi:10.1111/jcmm.70677)
Supplement: Supplementary file 4 — Table S2. [file JCMM-29-e70677-s003.pdf]

Supplementary Table 2. Modified hepatic activity index: Necroinflammation scores.

	Control	CCl_4	1μM COG133 + CCl_4	3 μM COG133 + CCl_4
**Interface hepatitis**	0	1,00 ± 0,00	0,50 ± 0,58	0,50 ± 0,58
**Portal inflammation**	0,25 ± 0,50	2,50 ± 0,58*	1,00 ± 0,00	1,25 ± 0,50
**Confluent necrosis**	0	5,50 ± 0,58*	0,50 ± 0,58	0,50 ± 0,58
**Lytic necrosis**	0	3,00 ± 1,15*	0,50 ± 0,58	0,50 ± 0,58
**Total score**	0,25 ± 0,50	12,00 ± 1,83*	2,50 ± 1,73	2,75 ± 1,26

Data are reported as mean ± SD and n=4 in each group. CCl_4, Carbon tetrachloride. COG133, ApoE mimetic peptide.
*, $p<0.05$ vs. control. Statistical analysis was performed by Kruskal-Wallis One Way Analysis of Variance on Ranks with all pairwise multiple comparison procedures by Tukey test.
